# A Spectrally Tunable Dielectric Subwavelength Grating based Broadband Planar Light Concentrator

**DOI:** 10.1038/s41598-019-48025-3

**Published:** 2019-08-13

**Authors:** Ameen Elikkottil, Mohammed H. Tahersima, M. V. N. Surendra Gupta, Rishi Maiti, Volker J. Sorger, Bala Pesala

**Affiliations:** 1 Academy of Scientific and Innovative Research, Chennai, India; 2 Council of Scientific and Industrial Research - Central Electronics Engineering Research Institute, Chennai, India; 3 Department of Electrical and Computer Engineering, George Washington University, Washington, DC USA

**Keywords:** Devices for energy harvesting, Photovoltaics, Solar energy and photovoltaic technology

## Abstract

Energy consumption of buildings is increasing at a rapid pace due to urbanization, while net-zero energy buildings offer a green and sustainable solution. However, limited rooftop availability on multi-story buildings poses a challenge for large-scale integration of photovoltaics. Conventional silicon solar panels block visible light making them unfeasible to cover all the surfaces of a building. Here, we demonstrate a novel dielectric grating based planar light concentrator. We integrate this functional device onto a window glass transmitting visible light while simultaneously guiding near infrared (NIR) portion of sunlight to edges of the glass window where it is converted to electricity by a photovoltaic cell. Gratings are designed to guide NIR region and realize polarization independent performance. Experimentally, we observe 0.72% optical guiding efficiency in the NIR region (700–1000 nm), transmitting majority of the visible portion for natural room lighting. Integrating solar cell at the window edge, we find an electrical conversion efficiency of about 0.65% of NIR light with a 25 mm^2^ prototype. Major losses are coupling and guiding losses arising from non-uniformity in fabrication over a large area. Such a functional window combining energy generation, natural room lighting and heat load reduction could mitigate urban heat island effect in modern cities.

## Introduction

The development of efficient optical concentrators to deliver a functional and economic solution for renewable power harvesting has focused mainly on solar photovoltaic and solar thermal power applications. Prominent methods utilize reflection (parabolic dish^1,2^), refraction (Fresnel lens^3–5^) or a combination of both reflective and refractive optics such as a compound parabola and prism^6^. Existing concentrators such as the Fresnel reflector, parabolic dish and light funnel^7^ require complex opto-mechanical frame works including moving lenses/mirrors to guide/manipulate the direction of light. However, these designs require sophisticated solar tracking, which make them bulky and expensive systems.

In contrast, advances in planar concentrators such as Luminescent Solar Concentrators (LSC)^8^ (either using dyes^9,10^ or quantum dots^11–13^), holographic concentrators^14–16^ and micro-optic concentrators^17–19^ demonstrate a low-cost and minimally tracking approach for solar light concentration. LSCs consist of dyes or quantum dots deposited on a transparent glass substrate^10^, which absorb incident solar energy over a certain band of wavelengths and reradiate in a different band (either down-shifted or up-shifted). Subsequently, the reradiated light is guided to the edges through the substrate. For instance, reported LSCs have demonstrated a power conversion efficiency of 2.85%^20^ (using silicon quantum dots over an area of 144 cm^2^), 2.8%^21^ (using CdSe quantum dots over an area of 15.4 cm^2^ with attached reflectors and solar cells) and an external quantum efficiency of ~3%^22^ (using CdSe and CdZnS quantum dots for a length of 100 cm). Major challenges of LSCs are degradation of dyes, self-absorption and low conversion efficiency^8,22–24^. Moreover, LSCs reradiate the light in all directions resulting in reduced coupling into the guided modes of the substrate. Additionally, in the current designs^8,25,26^ a relatively narrow band of the incoming solar radiation is harnessed (full width at half maximum, FWHM <200 nm).

A planar holographic concentrator is a potential technology to overcome these challenges. This system has shown power generation improvements of about 25% in solar cells^27^. However, holographic concentrators partially block visible light due to the placement of solar cells in the direct light path. Micro-optic concentrator is another class of planar concentrators in which a micro-lens and a diffuser is utilized to guide incoming sunlight to the waveguide. This technology can achieve higher optical concentration, as it utilizes multiple lenses to focus the light and couple to waveguides with rather low angular acceptance (<2°)^17^, thus requiring bulky and precise solar tracking infrastructure. Hotspot formation here results in accelerated degradation of solar cells^28^ and absence of wavelength selectivity causes thermalization losses thus reducing the overall conversion efficiency.

While conceptually promising, there are several challenges in the existing optical concentration technologies such as precise tracking, maintenance requirements, lower lifetime of opto-mechanical moving components, absence of spectrally selective light guiding, etc. Grating based designs are one of the approaches to realize a planar concentrator to overcome most of these challenges^29–31^. There a various High Contrast Grating (HCG) based designs such as aperiodic metasurfaces as a planar focusing element^32^, a two-dimensional metasurface as planar micro-lenses^30^ and a metal-based Fabry-Perot cavity solar cell^33^. In this work, we explore a subwavelength grating to realize a planar concentrator. Gratings diffract the incoming light into the glass substrate and can be designed to couple the light of desired wavelengths to first order transmission with highest efficiency by eliminating zero order transmission/reflection. Subwavelength gratings diffract the light at steeper angles over a broad wavelength range with high efficiency and this ensures guiding of desired wavelength band in the glass substrate. Grating based approach has the potential to eliminate the longer wavelengths (below bandgap) of sunlight reaching the solar cell which otherwise would not contribute to solar photovoltaic conversion and causes solar cell heating. The desired spectral selectivity of diffraction gratings can be tuned by optimizing the grating geometry parameters: grating period, duty cycle and thickness for a chosen material.

**Figure 1:**
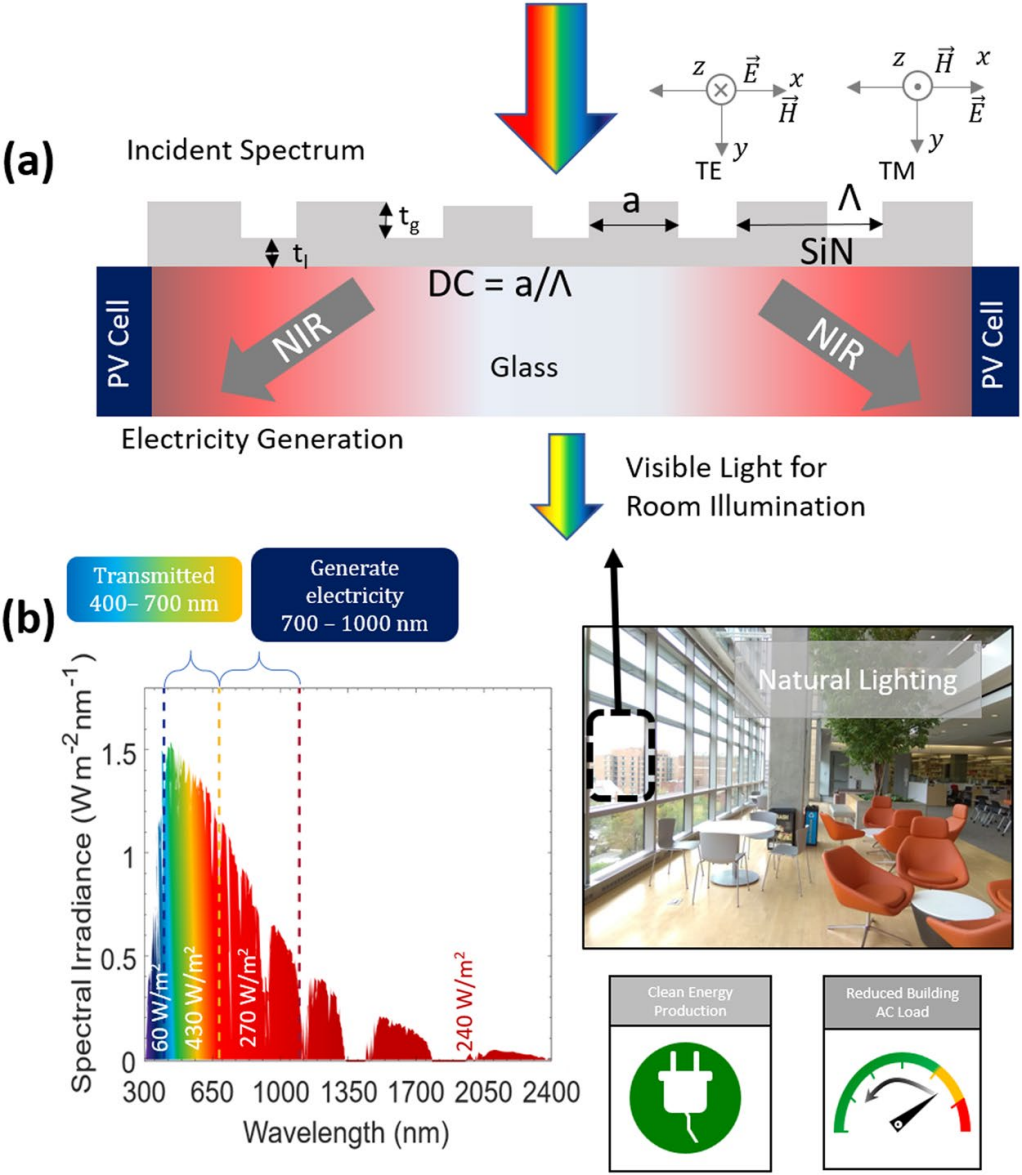
Proposed building integrated photovoltaics system based on a dielectric grating for broadband spectral filtering forming an optical concentrator. (**a**) Concept and schematic of the spectrally tunable grating based planar solar concentrator. The layer thickness is denoted as ‘t_l_’, grating thickness denoted as ‘t_g_’, the width of grating denoted as ‘a’, ‘Λ’ is the grating period and ‘a/Λ’ is the duty cycle of grating. (**b**) The distinct spectral regions in the solar irradiance: IR (λ > 1000 nm) with 240 W/m^2^, NIR in the wavelength range of 700–1000 nm with an irradiance of 270 W/m^2^ (operating range of the grating spectral concentrator shown as red arrows), the visible region from 400–700 nm with 430 W/m^2^ (transmitted) and λ < 400 nm corresponds to the UV region of AM1.5 G spectrum^48^.

Planar light concentrators offer significant benefits when used for building integrated photovoltaics. Modern dwellings such as skyscrapers are often covered with glass and hence trap the radiation thereby imposing additional heat load to the cooling systems and increase the temperature in the surrounding areas, commonly known as urban heat island effect. Recent studies claim that urban heat island effect contributes to 30% of climate warming^34^ and can lead to an annual mean air temperature rise up to 1–3 °C and as high as 12 °C^35^ in the evenings. Here, we propose and demonstrate a planar light concentrator using a silicon nitride subwavelength grating (Fig. 1) for conversion of NIR light to electricity, which has potential to be a smart power window. This planar concentrator is a multi-spectral light filter (Fig. 1(a)). It diffracts NIR radiation at steep angles into the window glass substrate where it is guided towards its edges and is converted to electricity using an edge-integrated solar cell. Simultaneously, it reflects thermally parasitic infrared radiation away from the building, which reduces the air conditioning load, while transmitting the visible light to enter the building, providing natural room lighting (Fig. 1(b)).

Our grating design is based on a selectively etched silicon nitride gratings on top of a glass substrate (Fig. 1(a)). Geometric design parameters defining spectral light guiding, reflection and transmission coefficients are grating period (Λ), duty cycle (DC), layer thickness (t_l_) and grating thickness (t_g_). For incident radiation with Transverse Electric (TE) polarization, the electric field is parallel to the grating bars while for Transverse Magnetic (TM) polarization, the magnetic field is parallel to the grating bars. Optimization enables the incident light of desired wavelengths to be diffracted into single transmission order^29^, thus effectively guiding the light to the window edges.

## Results

The dielectric grating concentrator consists of a transparent glass substrate with high refractive index coating (silicon nitride) followed by a grating of the same coating material (Fig. 1(a)). The grating is optimized to diffract the NIR portion of the incoming spectrum into the substrate. The angular space of the planar concentrator is defined by the grating diffraction condition:

1 $${\theta }_{m}={\sin }^{-1}(\frac{m\lambda }{{\rm{\Lambda }}\cdot {n}_{sub}}-\,\sin \,\theta )$$

where, ${n}_{{sub}}$: refractive index of the substrate, ${\theta }_{i}$: the incident angle, $\lambda$: wavelength of the incident light, ${\rm{\Lambda }}$: grating period, $m$: diffraction order, and ${\theta }_{m}$: diffraction angle.

Equation (1) shows that for a subwavelength grating, the higher diffraction orders contributing to reflection and transmission losses can be eliminated and guiding in the desired wavelength range is maximized. Effective guiding in the glass substrate is achieved by exciting first order diffraction modes that are guided inside the glass substrate towards the PV cell. For example, for a glass substrate (with refractive index of 1.5) and a grating period of 680 nm, the visible region (400–700 nm) diffracts in the angular range of 23.1°–43.3°. The diffraction angle for NIR wavelength range of 700–1000 nm is greater than the critical angle (41.8°) for glass/air interface, hence results in effective guiding into the glass substrate as desired here. The glass substrate acts as a multimode waveguide with different modes propagating at different angles (Eq. 1).

The maximum possible optical concentration of a HCG-based concentrator for a particular polarization is governed by^31,36:^

2 $${C}_{\max }=\frac{2{r}_{d}}{1-{r}_{dt}}\,\tan \,\theta $$

where, $\theta$: the diffraction angle for each wavelength, r_d_: the diffraction efficiency of incoming light to ±1 T (transmission order), r_dt_: diffraction efficiency +1 T to −1 T and vice versa.

However, Eqs 1 and 2 do not provide any insight regarding the diffraction efficiency of the grating structure except the angular distribution of different diffraction orders. Moreover, Eq. 2 does not show the relation of geometric concentration ratio and C_max_. Hence, FDTD simulations are carried out to obtain the optical guiding efficiency and transmission to map-out the geometric parameter space of the grating based light concentrator. We note that these simulations are also carried out with the actual grating profile obtained from cross-sectional Focused Ion Beam/Scanning Electron Microscope (FIB/SEM) images to evaluate the performance of the fabricated sample (Fig. 3(d)).

The choice of materials and geometry directly affects the grating performance. Rigorous Coupled Wave Analysis (RCWA) is used to optimize the grating structure and material. Materials such as silicon, titanium dioxide, silicon dioxide, zinc oxide and silicon nitride on a glass substrate are used in estimating the diffraction efficiencies and angles of the desired wavelength region. RCWA approximation considers the device structure as infinitely periodic but the actual structure is finite, hence we used the Finite Difference Time Domain (FDTD) method instead to evaluate the guiding efficiency (Fig. 3). However, FDTD simulation for actual dimension is computationally intensive and hence we have used a scaled down model (15 times) with the same geometric concentration ratio of 2.5 i.e. a substrate dimension of width 330 µm and thickness 66 µm. The device structure is optimized with silicon nitride as the grating material since the fabrication process is well developed compared to other materials for our structural dimensions and silicon nitride has comparatively high refractive index (>2)^37–39^, which allows for better optimization of diffraction, reflection and transmission^40^. In our case, the reflection in the NIR region, should be minimal to reduce optical loss. In addition to this, silicon nitride shows minimal optical absorption in the visible and NIR regions^41^, has low sensitivity to temperature variations^41^ and is widely used in state-of-the-art CMOS foundries^42^.

**Figure 2:**
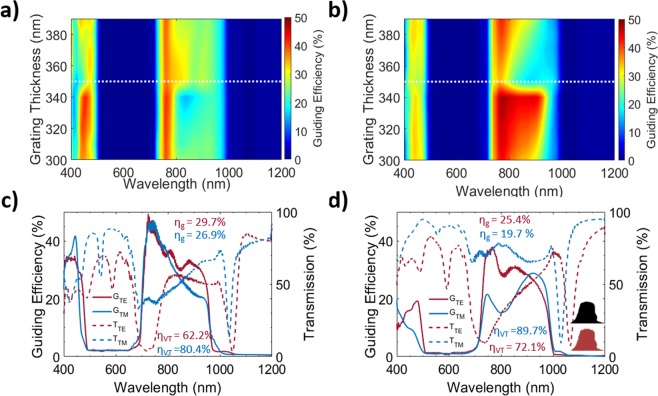
Numerical (FDTD) optimization of the grating parameters. (**a**,**b**) Optimization of grating parameters (DC, t_l_ and t_g_) for a polarization tolerant structure. Polarization tolerant planar concentrator guiding spectra as a function of grating thickness for TE and TM respectively at t_l_ = 90 nm. At a grating thickness of 350 nm (coarse optimization), the guiding efficiency spectrum for both TE and TM are similar. (**c**) TE & TM incidence guiding efficiency spectrum for grating parameters of Λ = 680 nm, DC = 0.5, t_l_ = 90 nm & t_g_ = 340 nm, the guiding efficiency (η_g_) for TE and TM are 29.7% and 26.9% in the wavelength range of 700–1000 nm respectively yielding an overall efficiency of 28.3%. (**d**) Guiding efficiency spectrum of the fabricated grating profile obtained from cross-sectional FIB/SEM is used to estimate the effect of non-rectangular profile and sidewall roughness (Λ = 680 nm, t_g_ = 280 nm, t_l_ = 120 nm & DC = 0.5). The inset shows the measured profile (top) and simulated profile (bottom) after profile extraction using image processing. The results show slight decrease in guiding efficiency compared to the ideal case due to the change in profile.

The visible light transmission efficiency (η_VT_ -Eq. S1) and guiding efficiency (η_g_ -Eq. S2) are the two key metrics used to evaluate the performance of planar concentrators (Fig. 2). These efficiencies are sensitive to the grating period, duty cycle and silicon nitride thickness. Visible light transmission accounts for the natural room lighting and guiding efficiency accounts for the generated electricity from NIR light. One-dimensional grating structures are mostly polarization dependent i.e. the guiding and reflection/transmission efficiencies vary with polarization of incident radiation. Hence, high guiding efficiency can be achieved in one of the polarizations, while it is lower for the other. However, sunlight is unpolarized in nature, so it is desired to have a minimal polarization dependence and hence an overall higher guiding efficiency can be achieved resulting in higher electrical output. Moreover, the guided wavelength and bandwidth can be tuned further to match any solar cell material bandgap towards improving the PV conversion efficiency (i.e. reduced non-radiative recombination) by adjusting the grating parameters. Here, we consider conventional silicon PV cells and aim for a guided spectral range of 700–1000 nm where the Si-PV conversion efficiency is high^43^.

Silicon nitride grating on glass can be tuned for polarization dependent or independent light guiding. In this paper, we mainly focus on minimally polarization dependent structures i.e. similar guiding spectra for both TE and TM polarized incidences, for energy conversion using solar cells. The simulation results shown in Fig. 2 considers surface normal incidence. The spectral grating thickness shows a parameter space where TE (Fig. 2(a)) and TM (Fig. 2(b)) spectra show matching guiding efficiency, with values of 29.7% and 26.9% for TE and TM incidences respectively (average of 28.3% efficiency) (Fig. 2(c)). The optimized grating parameters are DC = 0.5, t_l_ = 90 nm, t_g_ = 340 nm & Λ = 680 nm. The angular tolerance studies are carried out for the optimized design, the studies show that an angle tolerance of >100° (Fig. S8(a)) when the incidence angle is varied in the direction parallel (yz-plane in Fig. 1(a)) to the grating bars and 27° (Fig. S8(b)) in the perpendicular direction (xy-plane in Fig. 1(a)). A detailed temperature tolerance study is also carried out and the results are presented in section 10 of supplementary information, based on the study the variation in grating width and grating thickness will be maximum of 0.06 nm for an operating temperature variation of 50 °C. For a grating width/thickness variation of ±10 nm the combined guiding efficiency decrease is less than 1% (Fig. S9). Thus, ensuring the temperature stability of the planar concentrator in the actual operating conditions.

The grating profile has rectangular edges but the measured profile using FIB/SEM shows round edges, which affects the spectral guiding efficiency moderately. For the simulations of measured profile, a DC of 0.5 is considered as the grating widths are non-uniform across the 5 mm sample and the DC varies in the range of 0.31–0.69 (Fig. S3). The grating simulation parameters of Λ = 680 nm, t_g_ = 284 nm, t_l_ = 120 nm are used, which gives a minimal polarization dependence close to 935 nm wavelength which is observed in the experiments (Fig. 4(a)) as well. The simulation results for the rounded edges show slight TM to TE polarization discrepancy of 5.7% (Fig. 2(d)).

**Figure 3:**
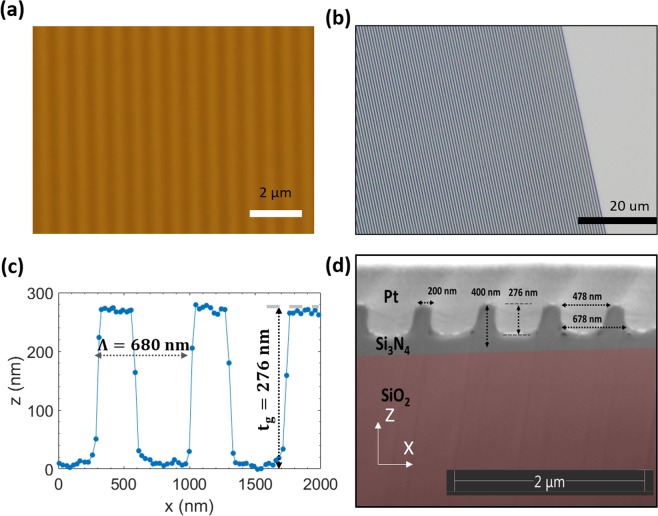
Grating fabrication and characterization. (**a**) Optical microscope image of electron beam lithography fabricated grating at lower magnification. (**b**) Optical microscope image of electron beam lithography fabricated grating at a higher magnification. Gratings are fabricated over a large area of 25 mm^2^ using electron beam lithography followed by reactive ion etching. (**c**) Atomic force microscope image of the electron beam fabricated sample after etching shows a grating thickness of 276 nm. (**d**) Cross-sectional FIB/SEM image of the fabricated sample, this grating profile and parameters are used in the simulation to estimate the effect of profile variation due to the etching process.

The dielectric gratings are fabricated with electron beam lithography over a large area of 25 mm^2^ (5 mm × 5 mm - fabrication process is given in Fig. S1). Reactive ion etching process is used as a pattern transfer onto the silicon nitride using electron beam resist polymethyl methacrylate (PMMA) as a soft mask and chromium layer as a hard mask. The grating period of the structure is relatively uniform over this area (Figs. 3(a) and 3(b)) and we obtain the height profile via atomic force microscope (Fig. 3(c)). We find that the sidewalls are relatively straight, however, the surface roughness on the grating has a root mean square value of 5.78 nm calculated from the results of atomic force microscopy (Fig. S6). To gain further insights into the profile of the grating, we have performed a cross-section using FIB milling and imaged it with a scanning electron microscope (Fig. 3(d)). Here, we find that the gratings have rounded edges, as compared to the targeted sharp edges, this affects the guiding efficiency by a few percentage and polarization tolerance, as discussed above (Fig. 2(d)).

**Figure 4:**
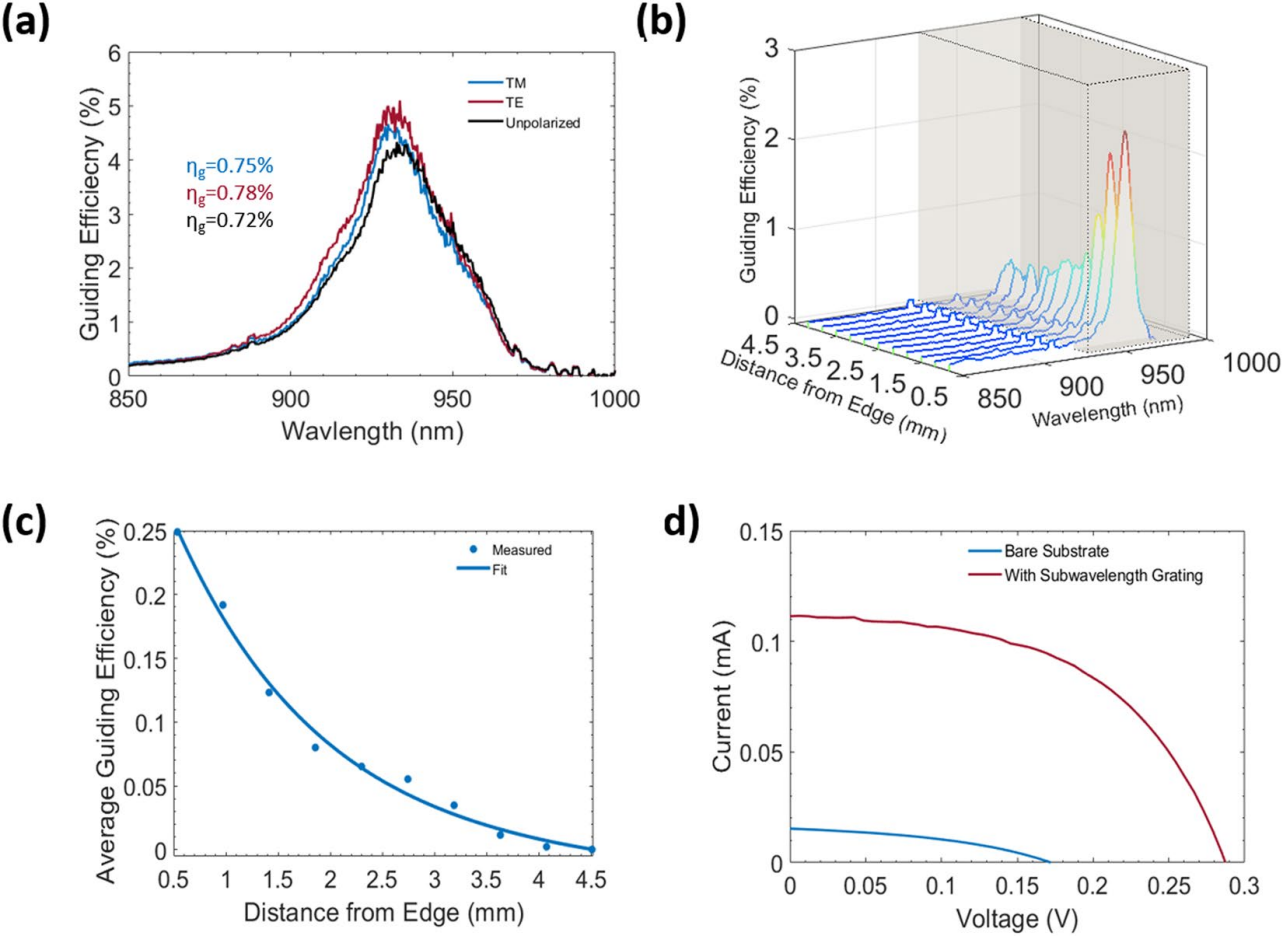
Optical characterization and performance evaluation of fabricated silicon nitride planar concentrator. (**a**) Guiding efficiency of the e-beam fabricated sample of dimensions 5 mm × 5 mm. The combined guiding efficiency for both the edges for TE, TM & unpolarized light are 0.78%, 0.75% & 0.72%, respectively in the wavelength range of 700–1000 nm (beam diameter of 4 mm). (**b**) Measured guided spectra as a function of distance from the edge close to the integrating sphere. (**c**) Average guiding efficiency as a function of distance from the edge close to the integrating sphere. The exponential decay in the guiding efficiency suggests guiding loss in the system (dots represent experimental data points and solid line represent curve fit using Eq. 3). (**d**) I–V curve with attached silicon solar cell on to one edge of the planar concentrator and the efficiencies reported are doubled accordingly. Reference sample is a glass coated with 400 nm thick silicon nitride.

Here, we show experiments demonstrating the conceptual functionality of the power window (Fig. 4). We observe a peak in the guiding spectrum at 935 nm (using measurement setup Fig. S4), which is independent of polarization as designed (Fig. 4(a)). The beam spot size used in this measurement is ~4 mm in diameter so that the scattering from the edges can be avoided. The polarization independency requires two uncoupled guided waves to be simultaneously excited^44^. This excitation happens only for particular combinations of grating parameters^45^. Moreover, the experimental results for TE and TM incidences show similar guiding spectrum (TE - 0.78%, TM - 0.75% & unpolarized - 0.72%). While these guiding efficiencies appear low as compared to the numerical results (Fig. 2(c)), they can be attributed to various losses in the system including material absorption, optical guiding loss, scattering loss due to roughness, non-uniformity of duty cycle and grating thickness across the sample. Even when there is low optical scattering in the substrate, longer optical path lengths can lead to higher scattering loss. For the guided modes, the optical path is longer for lower wavelengths and vice versa. Additionally, impurities in materials may cause additional optical scattering losses in a propagating mode. Moreover, the average visible light transmission for the fabricated sample is 64.9% and the transmission spectrum is shown in Fig. S12.

We use a technique similar to the cutback technique known from optoelectronics to investigate the effective optical loss (Fig. 4(b,c)). For effective loss evaluation, the guiding efficiency is measured as a function of distance from the edge of the substrate facing the integrating sphere with a beam diameter of ~1 mm. Indeed, we find a declining efficiency with distance to the sample edge. This can be understood from a multitude of effects such as material absorption, scattering and diffraction at silicon nitride layer/grating interface. These losses play a major role in the performance of the planar concentrator. We attempt to model the losses in the system with an assumption of mainly two components, a linear and an exponential loss component (Eq. 3). The linear component is mainly due to scattering centers because of defects and impurities^46^ of the substrate and the exponential component is due to the material absorption and guiding loss. From Beer- Lambert’s law, it is understood that the absorption has an exponential relation with the optical path length. The guiding efficiency also contributes to the exponential component. However, the material (glass) absorption losses are very low (average absorption coefficient in NIR is 2 × 10^−4^ mm^−1^ - Fig. S7). The guiding efficiency spectra (Fig. 4(b)) and average guiding efficiency (Fig. 4(c)) as a function of distance to the solar concentration edge show that the planar light concentrator has high losses. The measured data points and guiding efficiency model (curve fitting of the measured data points – Eq. 3) shown in Fig. 4(c) is used to estimate the losses.

3 $$g(x)={c}_{1}{e}^{-{c}_{2}x}-{c}_{3}x$$

Where, c_1_ = 0.37, c_2_ = 0.72 & c_3_ = 0.003 & x is distance in mm.

Fitting our distance-related optical powers at the sample edge with Eq. 3 (i.e. only considering absorption, guiding and scattering losses), we find a reduction of about 87% of the initial optical power due to losses (average propagation length of 2.5 mm). Out of these losses, the guiding loss arises from our design, which can be minimized through optimization. The main loss contributors in the exponential component of Eq. 3 are guiding and absorption losses; for every 1.4 mm of propagated distance the guiding efficiency becomes 1/e, i.e. the attenuation length. The absorption loss is almost negligible and major contribution is from the guiding loss i.e. out-coupling loss of the guided light in the glass substrate at the silicon nitride/glass interface. However, scattered light can also contribute to the loss. For the calculation (Fig. 4(c)), we have assumed that the main contributor to the scattering loss is from the linear term in Eq. 3. The scattering loss mainly arises from the glass substrate material impurities and defects, which can be minimized^47^. Thus, if at least the scattering losses were eliminated, the guiding efficiency would be 26% higher than the experimentally measured value i.e. 0.91% (Fig. 4(a)). Multiplication factor is obtained by the ratio of scattering excluded case to that of included for a distance 2.5 mm (i.e. x = 3 mm) using Eq. 3.

The performance of smart power window prototype is tested by integrating a silicon solar cell at the edge of the grating sample that captures the guided spectral power. The IV characteristics of prototype (Fig. 4(a)) shows that the short circuit current is 11 times more than that of the controlled sample. Similarly the open circuit voltage is 120 mV higher than that of the controlled sample. The controlled sample is a glass substrate coated with 400 nm silicon nitride (the same thickness of silicon nitride is used as in the grating structure). The silicon solar cell is diced into an area of 16 mm^2^ and covered with a copper sheet with black coating (to reduce unwanted reflections) to match the area of 7 mm^2^ equivalent to the edge area of the samples. We note that, this will introduce additional parasitic shading losses from the solar cell, which may adversely affect the performance of the system. This prototype shows a conversion efficiency of 0.65% under the solar simulator with an irradiance of 780 W/m^2^. The solar cell converts more than 80% of the incoming radiation, as there is lower thermalization loss in the guided spectrum at NIR wavelengths. If the scattering losses are minimized, the electrical efficiency in the NIR wavelength range can be increased to 0.82%. The planar concentrator scalability is discussed in detail in the supplementary information section 11. The results are promising, for a glass window of size 30 cm × 30 cm with a thickness of 15 mm (geometric concentration ratio of 10), the NIR guiding efficiency of >10% and electrical power conversion of 8% can be achieved.

**Table 1:** Solar cell parameters obtained with solar simulator under AM1.5 G illumination with 780 W/m^2^ for silicon PV cell placed only at one edge of the planar concentrator (device dimension of 25 mm^2^).

Sample	Optical Guiding Efficiency (%)	V_oc_ (V)	I_sc_ (mA)	P_max_ (mW)	Fill Factor (%)	Electrical Efficiency (%)
Control Sample (SiN layer on glass)	<0.01	0.17	0.01	<0.01	40	<0.01
Smart Power Window (SiN grating on glass)	0.72	0.29	0.11	0.02	52	0.65

In an ideal case (rectangular gratings with sharp edges), the average optical guiding efficiency can as high as about 28% and the corresponding electrical conversion efficiency in the NIR region (700–1000 nm) of about 17% (Fig. 2(c)). Even though, the experimental efficiencies are low compared with simulations as explained above, the lower efficiency is mainly due to the fabrication and material related imperfections; by further optimization of the fabrication process to realize large area uniform gratings and by reduction of the scattering losses and optimization of the grating design parameters, the optical guiding and electrical conversions of the smart power window can be further improved in the future.

## Summary

We demonstrated a transparent planar concentrator using a dielectric grating integrated onto a glass substrate. This system has a unique potential of generating electrical power from the NIR portion of the sunlight incident on the windows of modern buildings. The dielectric grating functions as a spectral filter on the incoming sunlight, which allows visible light to pass through the glass windows but guides the NIR light towards the glass edge for efficient PV conversion. We have demonstrated a prototype with an area of 25 mm^2^. The integrated device shows an optical guiding efficiency of 0.72% and solar cell conversion efficiency of 0.65% in the wavelength range of 700–1000 nm with an average visible light transmission of 64.9% in the wavelength range of 400–700 nm. Under the scenario of zero scattering losses, the guiding efficiency can reach to 0.91% and the solar cell conversion efficiency of 0.82%. The conversion efficiency of the device is not limited by the concept but with challenges in large area fabrication. As such, this planar concentrator prototype is well suited for building integrated photovoltaic applications and can become an integral part of future on-site electrical power generation, heating and cooling infrastructure for modern buildings. Such a device in principle can be fabricated over a large area using cost-effective techniques such as roll to roll interference lithography, stepper lithography and roll to roll nano-imprint lithography.

## Methods

### Design and simulation

Rigorous Coupled Wave Analysis (RCWA) based software from Grating Solver Development Co. (GSolver V5.2) is used to calculate the diffraction efficiencies of various material combinations and as a preliminary tool in choosing the materials. RCWA considers an infinitely periodic structure and yields only the diffraction efficiencies of various diffraction orders. Finite Difference Time Domain (FDTD) simulation methodology from Lumerical (FDTD Solutions 2018b R1 -v8.20.1634) is used to design a finite device and to obtain guiding efficiency at the edge of the substrate. The optimization process aims at maximizing the guiding efficiency followed by minimizing the polarization dependency. Simulations were carried out using a non-uniform mesh with a mesh accuracy of three. All the simulations consider two-dimensional geometry with PML and symmetric/anti-symmetric boundary conditions. The simulation results show Fabry-Perot resonance as the substrate thickness is very small. To eliminate this, smoothening is carried out on the obtained spectra. The material refractive index for silicon nitride utilizes the ellipsometer measured data (Fig. S2) and that for glass substrate is from Palik database.

### Fabrication

We developed a large area Electron Beam Lithography (EBL) process to fabricate silicon nitride grating on glass using the Raith VOYAGER EBL tool. After cleaning the glass substrate, it is coated with 400 nm of silicon nitride by using plasma enhanced chemical vapor deposition (Oxford PlasmaPro 100 PECVD) at a substrate temperature of 50 °C with gases such as silane (15 sccm) and nitrogen (12 sccm) at an inductively coupled plasma power of 500 W. Silicon nitride coated sample is characterized using surface profilometer (Bruker Dektak XT Profilometer) and ellipsometer (J. A. Woollam M-2000 DI Spectroscopic Ellipsometer) for obtaining thickness and refractive index parameters. The photoresist used for the EBL step (PMMA) is thin and shows poor selectivity during the reactive ion etch process of silicon nitride. An additional 30 nm thin chromium layer is sputter deposited (Denton Vacuum Discovery 550 Sputtering System) on the wafer that is used as the etch mask for silicon nitride. The optimized device geometry design (Fig. 2) is then used for the fabrication of the grating prototype. Using the EBL exposure, an area of 5 mm × 5 mm grating is fabricated on a 1 mm thick glass substrate. PMMA is spin coated at 4000 rpm for 45 seconds and subsequently baked at 180 °C for 2 minutes. The EBL tool uses an electron high tension of 30 keV with an aperture of 30 µm at a dosage of 400 µC/cm^2^. The chromium layer is then wet etched with PMMA as a masking layer using CR7. After removal of PMMA with acetone, RIE etch is carried out to etch the grating features on silicon nitride (CHF_3_ - 50 sccm and O_2_ - 5 sccm, RF power: 1000 W, operating pressure: 5 × 10^−6^ Torr using Unaxis 790 Reactive Ion Etcher). The etch depth is characterized by AFM (Bruker Dimension FastScan Atomic Force Microscope) and FIBSEM (FEI Helios FIB SEM).

### Optical and electrical characterization

The optical measurements are carried out to obtain transmission, reflection and guiding efficiency. The results for electron beam fabricated sample are shown in Fig. 4. The setup uses a fibre coupled halogen lamp from Ocean Optics HL 2000 followed by collimating optics with chromatic aberration correction. The light guided through the glass substrate is collected at the edge using an integrating sphere (ISP-R from Labsphere) and fed to the spectrometer (Ocean Optics USB2000).

Solar cell I–V characterization is carried out using Sol-3A solar simulator from Oriel Instruments at AM1.5 G with an illumination of 780 W/m^2^. I–V characteristics are measured using a Keithley 2440 source meter. The electrical measurements are carried out with solar cell at one edge. The result shown in Table 1 is doubled as in actual case solar cells will be integrated on both the edges.

## Supplementary information

A Spectrally Tunable Dielectric Subwavelength Grating based Broadband Planar Light Concentrator
