# Supplementary material for: A Spectrally Tunable Dielectric Subwavelength Grating based Broadband Planar Light Concentrator
Source: Sci Rep. 2019 Aug 13;9:11723. doi: 10.1038/s41598-019-48025-3 (PMC6692392; doi:10.1038/s41598-019-48025-3)
Supplement: Supplementary file 1 — A Spectrally Tunable Dielectric Subwavelength Grating based Broadband Planar Light Concentrator [file 41598_2019_48025_MOESM1_ESM.docx]

A Spectrally Tunable Dielectric Subwavelength Grating based Broadband Planar Light Concentrator

Ameen Elikkottil^1, 2, +,^ Mohammed H Tahersima^3, +^, Mvn Surendra Gupta^1,2^, Rishi Maiti^3^, Volker J. Sorger^3^, Bala Pesala^1,2, *^

^1^Academy of Scientific and Innovative Research, Chennai, India

^2^Council of Scientific and Industrial Research - Central Electronics Engineering Research Institute, Chennai, India

^3^ Department of Electrical and Computer Engineering, George Washington University, Washington DC, USA

*balapesala@gmail.com

Supplementary Information

1. Electron beam fabrication process flow

We initially optimized the process for electron beam lithography with PMMA as electron beam resist. As the substrate (glass) is non-conducting, substrate charging while writing was an issue and resulted in highly non-uniform grating. In order to mitigate the charging issue, a layer of chromium with thickness of 35 nm was used (etch selectivity between the deposited silicon nitride and chromium is >10). In addition, chromium serves as a hard mask, which is essential because PMMA is a soft mask and silicon nitride is highly resistant to etching process. The chromium layer was deposited using sputter technique.


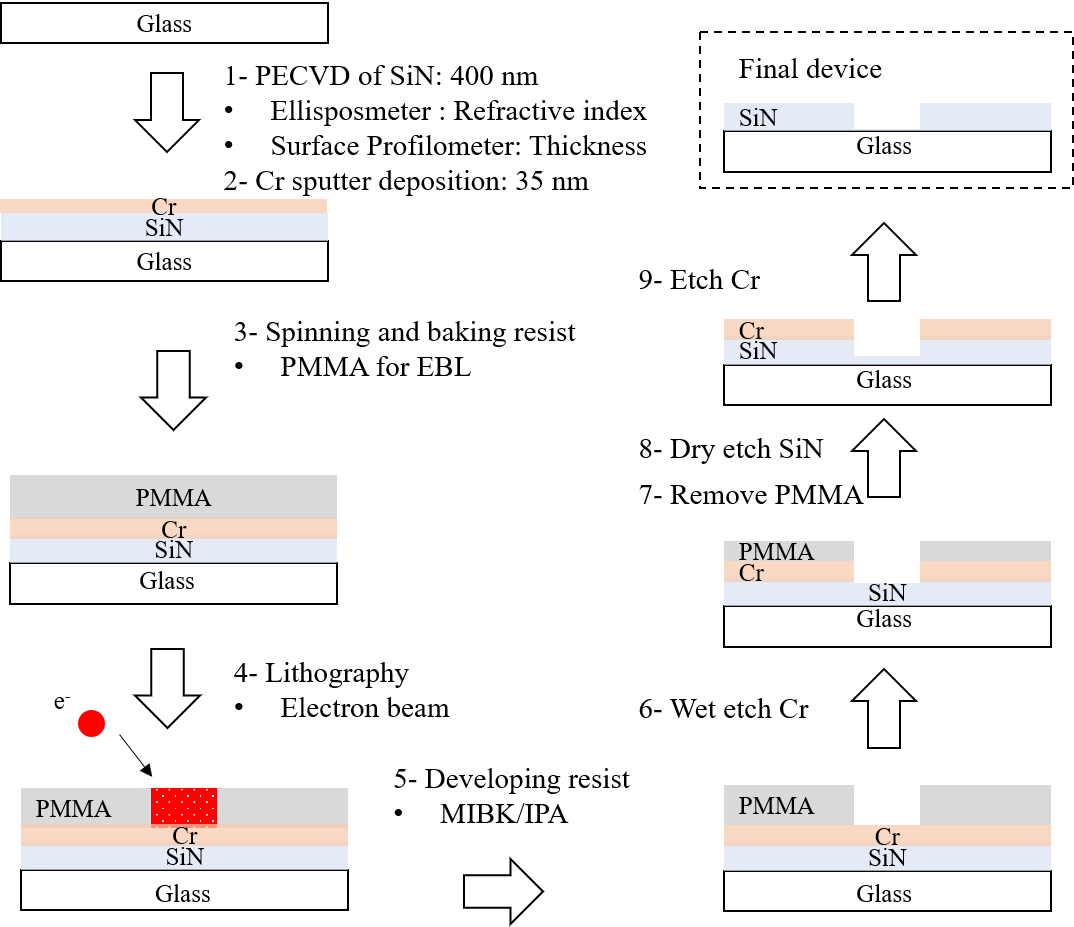


Figure S1. Process flow of grating fabrication using electron beam lithography and chromium etch mask.

1. Plasma Enhanced Chemical Vapour Deposition (PECVD) of silicon nitride

PECVD was used to deposit SiN on a glass substrate at a temperature of 50˚C with 119 nm per minute deposition rate. Silane (15 sccm) and nitrogen (12 sccm) gases are used for the silicon nitride deposition at an inductively coupled plasma power of 500 W. The refractive index and thickness of the sample is verified using ellipsometry and surface profilometry respectively. The measured thickness of the silicon nitride layer is 400± 40 nm over 4” wafer.

1. Grating fabrication results – Scanning Electron Microscope (SEM)


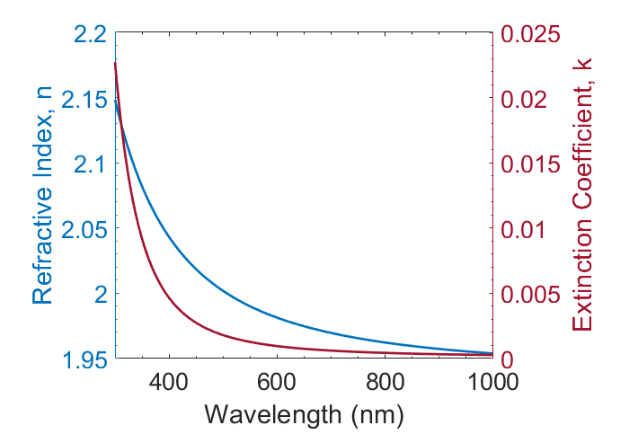


Figure S2. Refractive index profile of PECVD coated silicon nitride on glass substrate measured using ellipsometer.


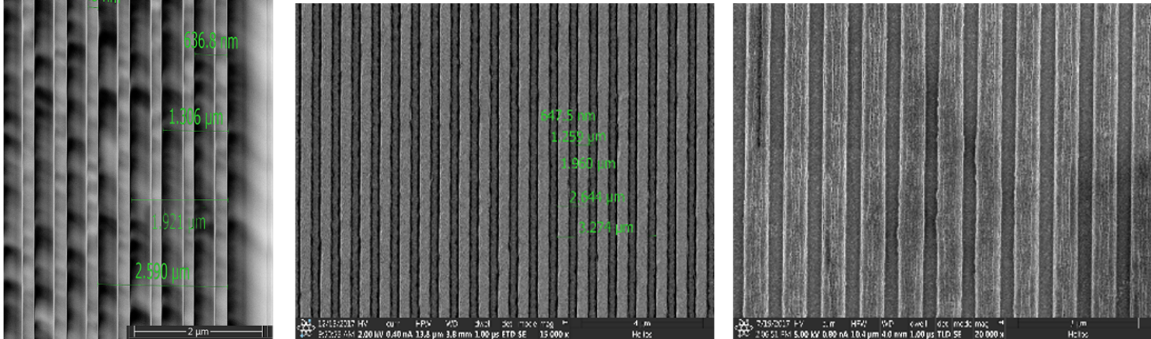


FigureS3. SEM images of the electron beam lithography fabricated grating structure.

The top view SEM image of the fabricated gratings (Fig. S3) shows that the gratings have surface roughness, which arises due to the etching process. This can induce scattering losses and reduce the optical guiding efficiency. The process of etching is carried out with reactive ion etching using CHF_3_ (50 sccm) and O_2_ (5 sccm) at 1000 W RF power under a pressure of 5x10^-6^ Torr using Unaxis 790 Reactive Ion Etcher. This process yields an average etch rate of 0.83 nm/sec for silicon nitride for the desired pattern.

1. Guiding efficiency and transmission calculation

Visible light transmission (η_VT_– eqn. S1)^1,2^ gives the amount of visible light transmitted through the substrate for indoor lighting. The guiding efficiency (η_g_ – eqn. S2) is the parameter used to evaluate the amount of light guided through the substrate towards the edge. These two planar concentrator performance metrics are given by:

$\eta_{\text{VT}}=\frac{\int_{400}^{700} I(\lambda)T(\lambda)P(\lambda)d\lambda}{\int_{400}^{700} I(\lambda)P(\lambda)d\lambda}\times100\%$ (S1)

$\eta_{\text{g}}=\frac{\int_{400}^{700} I_{g}(\lambda)T(\lambda)d\lambda}{\int_{400}^{700} I(\lambda)d\lambda}\times100\%$ (S2)^1^

Where, I (λ): Incident spectrum, I_g_ (λ): Guided spectrum, T (λ): Transmitted spectrum, P(λ): Human eye response

1. Optical characterization setup

| 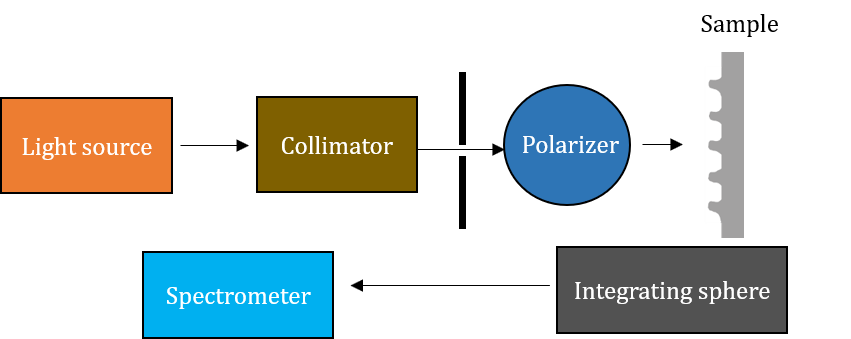 |
| --- |
| Figure S4. Optical characterization setup a) schematic of the characterization set up b) In-house built characterization setup. |

The optical setup (Fig. S4) consisting of a fibre coupled halogen light source HL 2000 from ocean optics, is used for measurement of guiding efficiency through the substrate. The light source is coupled to an objective lens (collimator) with 4x magnification using SMA fibre. An iris is used to control the beam spot size followed by a polarizer to control the polarization of the incident beam. Integrating sphere (ISP-R from Labsphere) connected to a USB2000 spectrometer from Ocean Optics using an optical fibre is used to collect and measure the spectrum of guided light.

1. Profile extraction from fib/sem image

| 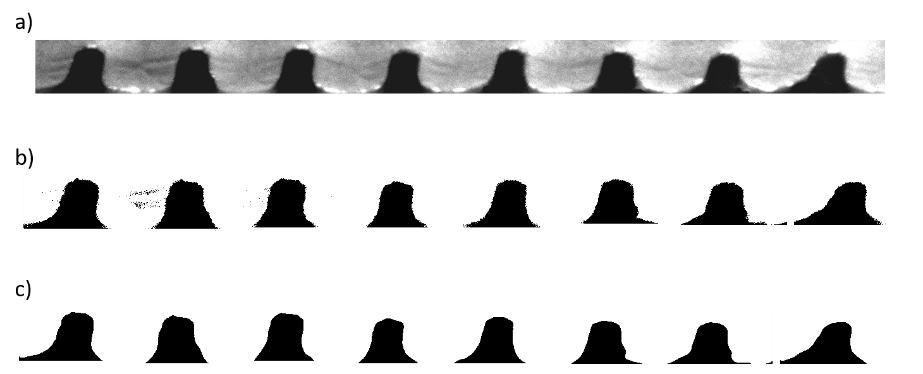 |
| --- |
| Figure S5. Profile extraction for simulation a) FIB/SEM image of the electron beam fabricated sample b) Binary image profile after background removal c) Profile after removal of noise and edge smoothening. |

The raw FIB/SEM image (Fig. 3(d)) is processed and resized (Fig. S5(a)). A running window filter of fixed width (same as grating period) is used to select the individual profile and subsequently the profiles are converted into binary images (Fig. S5(b)). However, the obtained binary images are noisy with rugged edges (Fig. S5(c)). The edge smoothened and noise removed profiles (Fig. S5(c)). Matlab^®^ is used for these image processing and profile averaging. The sixth profile in Fig. S5(c) is used in simulations as it shows minimum polarization dependency.

1. Atomic force microscope based surface roughness calculation

In order to calculate the Root Mean Square (RMS) of surface roughness over the flat surface of the grating, that is crests (marked with blue dotted curves in Fig. S6(a)) and troughs (marked with red dotted curves in Fig. S6(a)) without considering the sidewall roughness, we have used the atomic force microscope result. In the process, we have selected and appended all the crest points (Fig. S6(b)) followed by negative offsetting of these points with the mean. Similar calculation is carried out for the trough points (Fig. S6(c)). Then both these trough and crest points are appended for the calculation of RMS. The RMS value for the appended points is 5.78 nm.

| 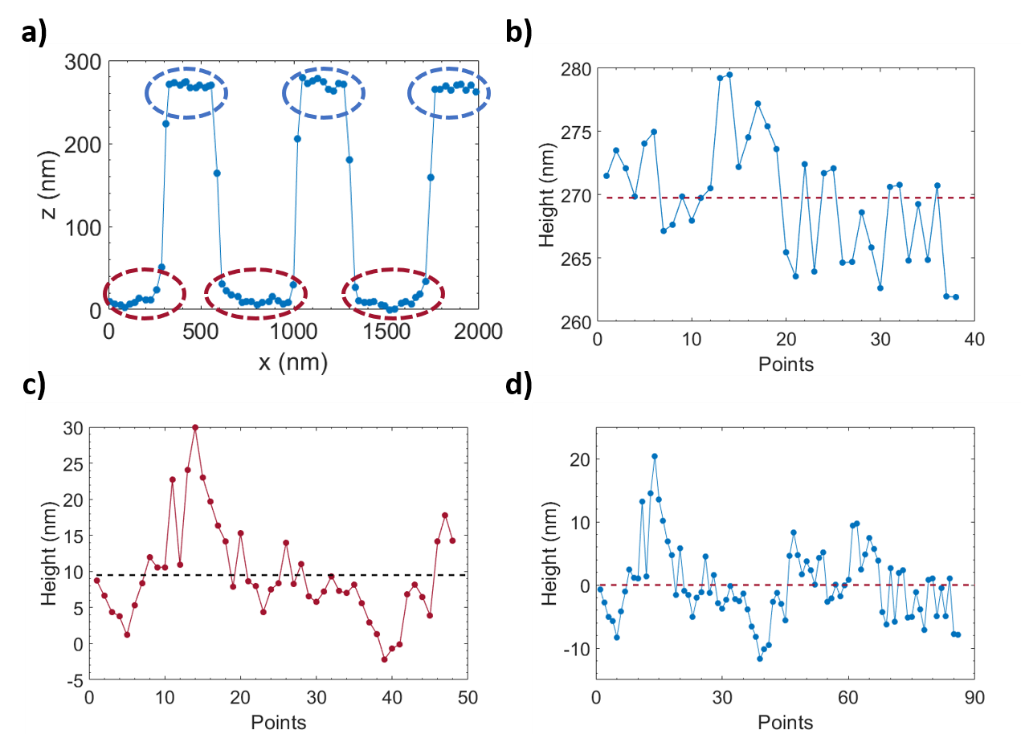 |
| --- |
| Figure S6. Root mean square calculation a) AFM image of the electron beam fabricated sample showing the crests (blue) and troughs (red) b) Appended points of the crest with mean value (dotted line) c) Appended points of the trough with mean value (dotted line) d) Both crest and trough values appended together after subtracting the corresponding means. |

1. Substrate absorption calculation

Extinction coefficient (k) is used to calculate absorption coefficient for the glass substrate (Sumita BK7 commercial glass) used in the fabrication of planar concentrator. The average absorption coefficient (Fig. S7) is 2 x 10^-4^ mm^-1^, which is negligible in the NIR wavelengths (700-1000 nm) as compared to the guiding loss.

| 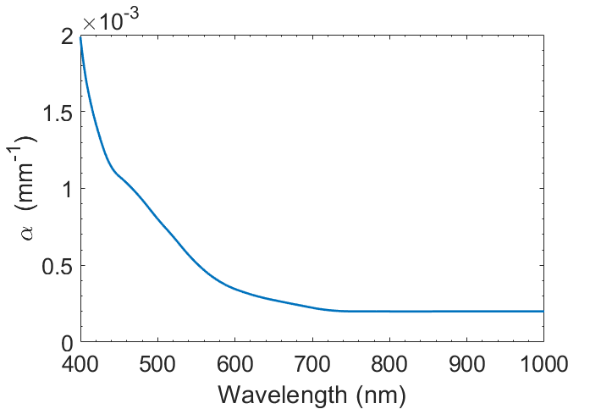 |
| --- |
| Figure S7. The absorption coefficient of the glass substrate used for planar concentrator fabrication. |

1. Angular study for the optimized grating structure

The angular studies are a carried out using FDTD simulation with 2D and 3D simulation geometry for perpendicular and parallel incidence angle variations with respect to the grating bars direction respectively. A non-uniform meshing with mesh accuracy of 2 is used in simulations. These studies are carried out for the optimized polarization tolerant planar concentrator design (geometric concentration ratio of 2.5) with grating parameters: grating period of 680 nm, duty cycle of 0.5, grating thickness of 340 nm and layer thickness of 120 nm. The angular study (shown in Fig S8), shows that an angular tolerance >100° (with a resolution of 1°) can be achieved for the planar light concentrator for the incidence angle varying in the parallel direction (Fig. S1(a)) of grating (yz-plane in Fig. 1(a)) and 27° in the perpendicular direction (Fig. S1(b), the dotted line shows the 50% guiding compared to the normal incidence) of grating (xy-plane in Fig. 1(a)). The planar concentrator can be aligned in such way that the gratings are in the east west directions to achieve higher operating hours during day time to capture maximum sunlight.

| 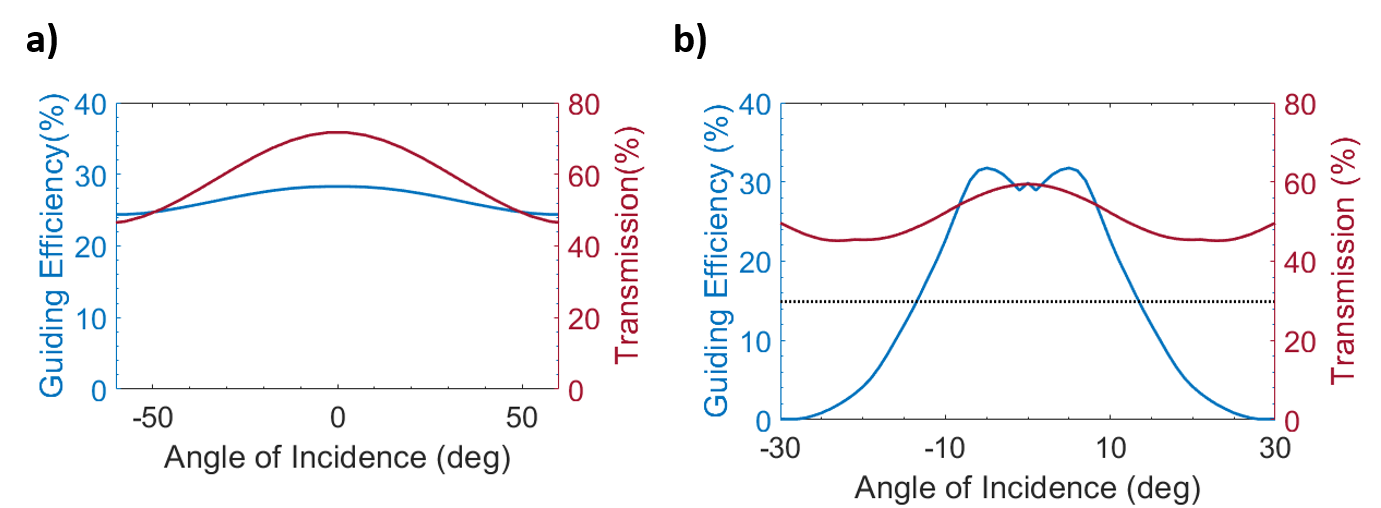 | |
| --- | --- |
| Figure S8. The angular study for the optimized grating with grating period 680 nm, duty cycle of 0.5, grating thickness of 340 nm and layer thickness of 120 nm. a) Angular tolerance when the incident angle is varied across parallel direction and shows a tolerance >100° in with decrease of efficiency less than 4%. b) Angular tolerance when the incident angle is varied across perpendicular direction and shows ±13.5° with 50% decrease in efficiency. | |

1. Temperature tolerance of the optimized design

The temperature tolerance studies are carried out to understand the effect of variation in grating width and grating thickness on the guiding efficiency. The FDTD simulations for temperature tolerance study considers a 2D simulation geometry with geometric concentration ratio of 2.5 and a non-uniform meshing with mesh accuracy of 2. The planar concentrator based window is expected to operate in a wide-range of temperature conditions resulting in a large temperature difference greater than 50ºC (ΔT). Based on the thermal expansion coefficients for silicon nitride (3.27 × 10^−6^ /ºC)^3^ both grating with and thickness variation is 0.06 nm. The grating width and the thickness are varied from 330 nm - 350 nm with a step of 1 nm. The results shown in Fig. S9 shows that the combined guiding efficiency (average of both TE and TM) changes less than 1% (Fig. S1(a)) for grating width variation of ±10 nm. Similarly, for a grating thickness variation of ±10 nm the combined guiding efficiency changes by less than 1%.

| 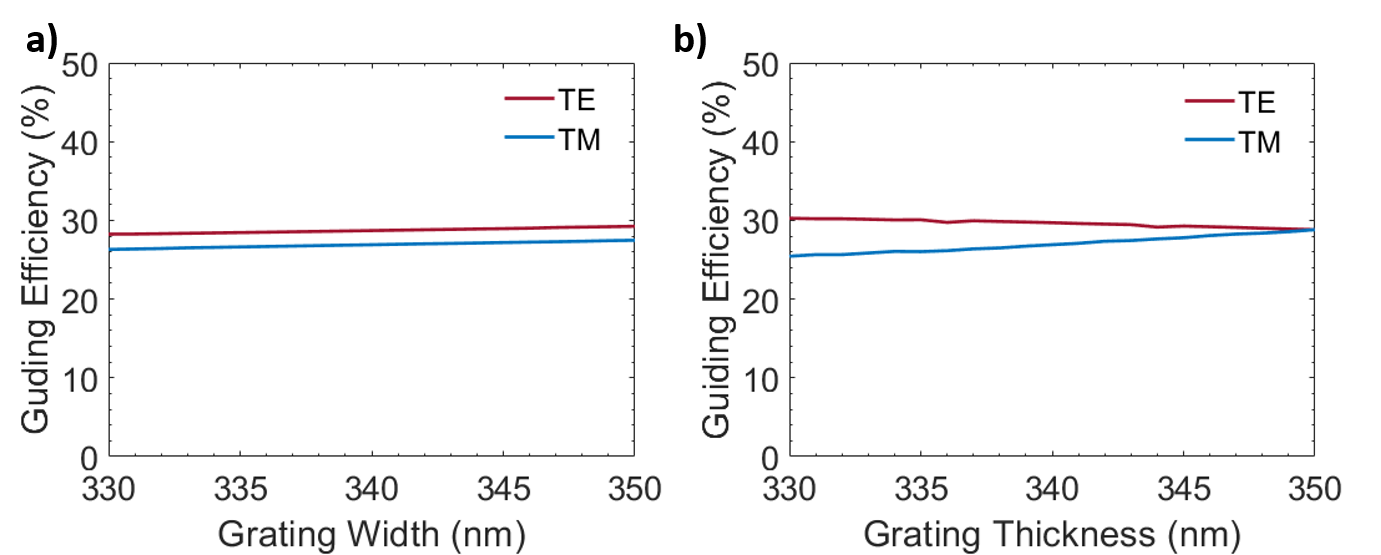 |
| --- |
| Figure S9. The temperature study for the optimized grating with grating period 680 nm, duty cycle of 0.5, grating thickness of 340 nm and layer thickness of 120 nm. a) Guiding efficiency with respect to grating width shows a variation of less 1% in efficiency for a change in width of ±10 nm b) Guiding efficiency with respect to grating thickness shows a variation of less 1% in efficiency for a change in thickness of ±10 nm. |

1. Planar concentrator scalability studies

A detailed study for the scalability is carried out using FDTD simulations. The simulation uses a 2D geometry with a non-uniform meshing of mesh accuracy two. The geometric concentration ratio considered in the study are 2.5, 5, 7, 10 and 15. Based on the simulation results (Fig. S10), for a geometric concentration ratio of 10, greater than 10% combined guiding efficiency (average of both TE and TM) can be achieved. Thus, considering a geometric concentration ratio of 10, for a glass thickness of 15 mm, a window size of 30 cm x 30 cm can be built with a NIR guiding efficiency of 10% (Fig. S10). For a silicon cell, this NIR guiding efficiency yields an electrical conversion efficiency of 8% in the considered NIR region (700-1000 nm). The design can be further optimized to achieve higher performance for a geometric ratio of 10. Scaled design (30 cm x 30 cm) can be realized using fabrication techniques such as DUV/stepper lithography, nano-imprint lithography, displacement Talbot lithography and laser interference lithography.

| **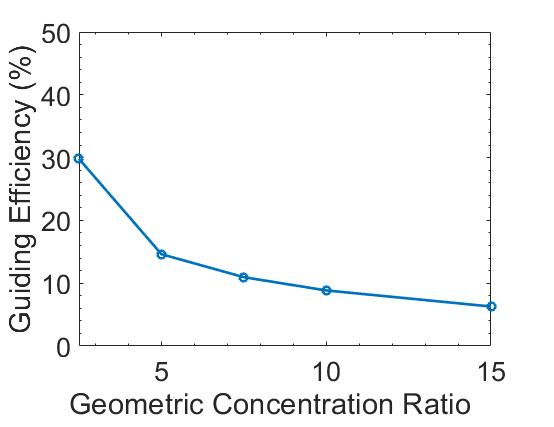** |
| --- |
| Figure S10. Variation of combined guiding efficiency (average of TE and TM) with respect to increasing geometric concentration ratio. |

1. Polarization dependent structure

| 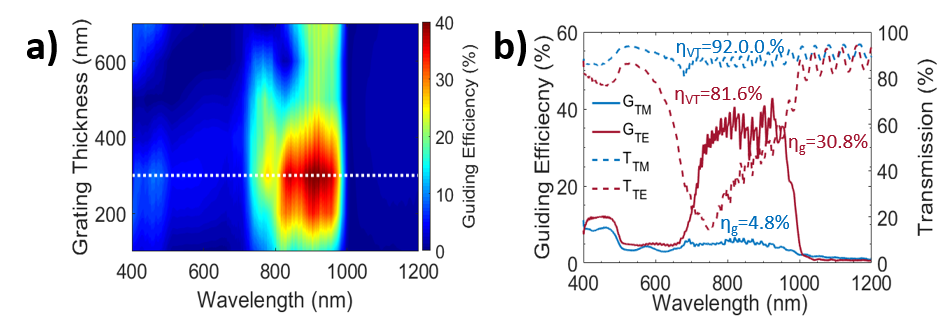 |
| --- |
| Figure. S11. Numerical (FDTD) optimization of the grating parameters. a) Optimization of grating parameters (DC and t_g_) for a polarization dependent structure. TE incidence guiding efficiency spectrum as a function of grating thickness for Λ=680 nm & DC=0.3. Similar studies are carried out for different duty cycles and DC=0.3 gives better result. It can be inferred that for a grating thickness of 300 nm (white dotted line at t_g_=300 nm) the design shows higher guiding efficiency (including both the sides). b) Optical guiding and transmission spectra of the planar concentrator for grating parameters Λ=680 nm, DC=0.3, t_l_ =0 nm & t_g_=300 nm for TE and TM incidences. The guiding efficiency (η_g_) for TE and TM incidence is 30.8% and 4.8% respectively and averages to 17.8% in wavelength range of 700-1000 nm. The results show visible light transmission (η_vt_) of 81.6% and 92.0% in the range of 400-700 nm for TE and TM incidences, respectively. |

In polarization dependent design, the guiding efficiency (η_g_=30.8%) is maximized for TE incidence as shown in Fig. 2(a). The grating period and duty cycle are constant, i.e. Λ=680 nm and DC=0.3, respectively while the grating thickness is varied from 100 nm to 700 nm. TE incidence guiding efficiency is maximum at a grating thickness of 300 nm. However, TM incidence guiding efficiency for the same design is minimal (η_g_=4.8%), this can potentially find application in spectrally tuneable polarized beam splitter (Fig 2(b)). However, the proposed design focuses on minimal polarization dependent structures.

1. Transmission measurement of the electron beam fabricated sample

| 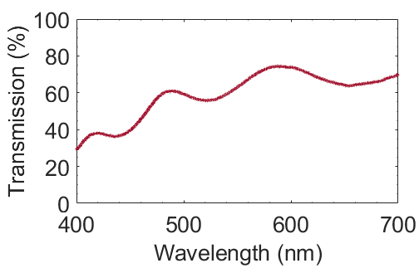 |
| --- |
| Figure. S12. Transmission spectrum of the electron beam fabricated subwavelength grating sample. |

The transmission spectrum is obtained using the setup shown fig. S4 with slight modification in positioning of the integrating sphere. The integrating sphere is placed such that the transmitted light through the sample is collected. The calculated η_VT_ for the sample is for the sample is 64.9%.

References:

1. Wu, K., Li, H. & Klimov, V. I. Tandem luminescent solar concentrators based on engineered quantum dots. *Nat. Photonics* **12,** 105–110 (2018).

2. Lunt, R. R. & Lunt, R. R. Theoretical limits for visibly transparent photovoltaics Theoretical limits for visibly transparent photovoltaics. **043902,** (2013).

3. Tien, C.-L. & Lin, T.-W. Thermal expansion coefficient and thermomechanical properties of SiN _x_ thin films prepared by plasma-enhanced chemical vapor deposition. *Appl. Opt.* **51,** 7229 (2012).
